# Peripheral Immune Cell Gene Expression Predicts Survival of Patients with Non-Small Cell Lung Cancer

**DOI:** 10.1371/journal.pone.0034392

**Published:** 2012-03-29

**Authors:** Andrew V. Kossenkov, Noor Dawany, Tracey L. Evans, John C. Kucharczuk, Steven M. Albelda, Louise C. Showe, Michael K. Showe, Anil Vachani

**Affiliations:** 1 The Wistar Institute, Philadelphia, Pennsylvania, United States of America; 2 Division of Hematology/Oncology, Department of Medicine, University of Pennsylvania School of Medicine, Philadelphia, Pennsylvania, United States of America; 3 Division of Thoracic Surgery, Department of Surgery, University of Pennsylvania School of Medicine, Philadelphia, Pennsylvania, United States of America; 4 Division of Pulmonary, Allergy, and Critical Care Medicine, Department of Medicine, University of Pennsylvania School of Medicine, Philadelphia, Pennsylvania, United States of America; IPO, Inst Port Oncology, Portugal

## Abstract

Prediction of cancer recurrence in patients with non-small cell lung cancer (NSCLC) currently relies on the assessment of clinical characteristics including age, tumor stage, and smoking history. A better prediction of early stage cancer patients with poorer survival and late stage patients with better survival is needed to design patient-tailored treatment protocols. We analyzed gene expression in RNA from peripheral blood mononuclear cells (PBMC) of NSCLC patients to identify signatures predictive of overall patient survival. We find that PBMC gene expression patterns from NSCLC patients, like patterns from tumors, have information predictive of patient outcomes. We identify and validate a 26 gene prognostic panel that is independent of clinical stage. Many additional prognostic genes are specific to myeloid cells and are more highly expressed in patients with shorter survival. We also observe that significant numbers of prognostic genes change expression levels in PBMC collected after tumor resection. These post-surgery gene expression profiles may provide a means to re-evaluate prognosis over time. These studies further suggest that patient outcomes are not solely determined by tumor gene expression profiles but can also be influenced by the immune response as reflected in peripheral immune cells.

## Introduction

Lung cancer is the most common cause of cancer mortality worldwide, accounting for 157,300 cancer deaths in the United States alone in 2010 [1]. The overall five-year survival for lung cancer is 16%, and prognosis is strongly associated with the disease stage at diagnosis [2]. Non-small cell lung cancer (NSCLC) accounts for 80% of all lung cancer cases.

Treatment protocols and prognostic assessments of patients with NSCLC are based primarily on TNM stage. Surgical resection for early stage disease (Stage I, II, and some Stage III) remains the standard of care. Unfortunately, 30–60% of patients (depending on stage) will develop a recurrence and die of their disease, leading to a 5-year survival rate of 35–70% for patients after resection. Clearly there is an unmet need for additional prognostic factors for a more informed process of treatment.

There is significant heterogeneity in clinical outcomes for patients with early stage NSCLC and the basis is unknown. Several previous studies focused on gene expression in surgically excised tumors to identify prognostic signatures were recently reviewed [3], [4]. None is yet approved for clinical application.

We previously showed that patients with NSCLC have significant gene expression changes in their PBMC which provide useful diagnostic markers (5) and this PBMC cancer signature is reduced or eliminated in a subset of patients retested after tumor resection [5]. Since the changes in PBMC gene expression are a reflection of the interactions of the tumor and the immune system, we have now analyzed our gene expression data and demonstrate a signature associated with overall survival. We also show that some PBMC genes associated with survival change their expression in samples taken after tumor resection and might provide an additional indicator of recurrence.

## Materials and Methods

### Study population

A total of 137 patients with newly diagnosed, histopathologically confirmed, non-small cell lung cancer (NSCLC) were recruited from the University of Pennsylvania Medical Center during the period 2003 through 2007. Written informed consent was received from all participants involved in the study and samples were collected with approval of both University of Pennsylvania IRB and Wistar IRB. For this analysis, only subjects with Stage I-IIIA NSCLC who underwent surgical resection with curative intent were included. Exclusion criteria included sub-lobar resection, positive resection margins, and death within 30 days of surgery. This resulted in the inclusion of 108 subjects in this analysis (**Table S1**). All participants had blood collection prior to surgery and 15 of the 108 patients also had blood collected after surgery. **Table 1** summarizes the major prognostic parameters as identified by the National Cancer Comprehensive Network (NCCN) for our study population. Clinical outcome and survival information was obtained via chart review, phone contact, or from the Social Security Death Index (SSDI).

**Table 1:** NCCN factors tested for association with survival.

Variable	Variable Details	Univariate Cox p-value	HR [95% CI]	Multivariate Cox p-value/HR
Stage	Stage.I = 66, Stage.II/III = 42	0.002	2.49 [1.39–4.47], Stage.II/III vs Stage.I	**0.002, HR = 2.47**
Age	min = 45 yo, med = 68 yo, max = 87 yo	0.03	1.04 [1.00–1.07], per year increase	**0.03, HR = 1.04**
Gender	F = 55, M = 53	0.74	1.10 [0.62–1.97], Female vs Male	not tested
Race	AA = 9, Caucasian = 99	0.32	2.06 [0.50–8.49], AA vs Caucasian	not tested
COPD	present = 50, absent = 54	0.2	1.47 [0.81–2.67], present vs absent	not tested
Histology	AD = 67, LSCC = 34	0.4	1.31 [0.70–2.44], AD vs LSCC	not tested
Tobacco use	previous = 87, current = 15	0.99	0.99 [0.44–2.23], previous vs current	not tested
Adjuvant Chemo	no = 52, yes = 34	0.19	1.58 [0.80–3.14], no vs yes	not tested
Pack years	min = 0 py, med = 40 py, max = 188 py	0.87	1.00 [0.99–1.01], per pack increase	not tested

Variable details show number of patients for categorical variables and minimum (min), median (med) and maximum (max) values for continuous variables. HR = hazard ratio, CI = confidence interval, F = female, M = male. AD = lung adenocarcinoma, LSCC = lung squamous cell carcinoma. AA = African American, yo = years old, py = pack years.

### PBMC Collection and Processing

Blood samples were drawn at the University of Pennsylvania Medical Center in two “CPT” tubes (Becton Dickinson). Peripheral blood mononuclear cells (PBMC) were isolated within 90 minutes of blood draw, washed in PBS, transferred into RNAlater (Ambion), and then stored at 4°C overnight before transfer to −80°C. RNA purification was carried out using TriReagent (Molecular Research), as recommended, and controlled for quality using the Bioanalyzer. Only samples with 28S/16S ratios of >0.75 were used for further studies. A constant amount (400 ng) of total RNA was amplified, as recommended by Illumina, for gene expression analysis.

### Microarray preprocessing

Samples were processed and hybridized to the Illumina WG-6v2 human whole genome bead arrays. All arrays were processed at the Wistar Institute Genomics Facility as previously described [6] and then gene expression values were log_2_ transformed for further analysis. The array data used for these studies are publicly available in the GEO database through the accession number GSE13255. **Table S1** lists the 108 subjects used for analysis.

### Statistical Methods

#### Risk factor analysis

Univariate Cox regression analysis was performed to test the association of clinical risk factors and individual gene expression with overall survival. Multivariate Cox regression was used to test the association of a combination of factors. Statistical significance was defined as *P*<0.05. False discovery rate for gene association with overall survival was determined according to Storey et. al. [7]. Association of overall survival with tumor stage was done both by treating stage as a continuous variable and as two groups: stage I and a combined stage II plus III. The results were significant for both approaches with the univariate and multivariate regression analyses. Because the number of stage II tumors was small (14 patients) and even smaller when the data was split into training and independent validation sets, we decided to combine stage II and III to have a larger group size in the validation step. We used the combination of stage II and III throughout the manuscript for consistency.

#### Kaplan-Meier curves

Kaplan-Meier curves were plotted using Matlab v7.2 based on censored survival data. Patients were stratified by factor median unless stated otherwise.

#### Gene panel selection

To identify a set of genes associated with overall survival we split the 108 sample dataset into training and testing sets of equal size (54 random samples in each). The training set was first analyzed to identify an outcome-informative gene panel by identifying probes with the lowest mean univariate Cox regression p-value across 50 tests of 40 random samplings (75% of the training set). The top 100 genes were then used for multivariate Cox regression with L1 and L2 penalized estimations [8]. The final λ1 and λ2 hyperparameter pair selected gave the best performance using 10-fold cross-validation on the training set. This method results in a model consisting of N genes that have non-zero regression coefficients c_1_…c_N_. Those coefficients along with corresponding gene expression data for any patient *i* (X_1i_… X_Ni_) were used to calculate a gene prognostic score (GPS) for the patient as follows: GPS = c_1_X_1j_+…+c_N_X_Nj_.

#### Independent validation of the predictive gene panel

The 54 patients set aside and not used for predictive gene selection were used as an independent test set for evaluation of the model developed on the training set. Significance of the GPS as a prognostic factor on the external validation set was estimated by univariate Cox regression. Independence of the GPS and Stage factors were tested by multivariate Cox regression.

#### Testing robustness of the gene panel selection

To show that the significance of independent validation (performance) of the GPS model on the testing set did not depend on the particular training-testing data split, we performed 100 random splits and showed the quality of separation between low and high-risk patients in testing sets is independent of any particular data split. (**Figure S1**)

#### Testing efficacy of prognostic indicators

Efficacy of the prognostic indicators was tested by comparing hazard ratios and concordances. Hazard ratios were calculated between high and low risk groups. Concordance was estimated using the R package *clinfun* [9].

#### Gene enrichment analysis

Testing for biological functions and pathways overrepresented in a gene list was done using DAVID software [10] with thresholds set at false discovery rate (FDR) FDR<20% and enrichment >2 fold. For a gene list with high false discovery rate (52% for 1704 genes associated with overall survival) we report only results that pass a threshold of FDR<1% and also show significant enrichment by GSEA algorithm [11], [12] with FDR<25% cutoff.

#### Enrichment of immune cell-type specific genes

We obtained a list of genes specifically expressed in 8 categories of immune cell types, including T cells, B cells, NK cells, Dendritic cells, Monocytes, Neutrophils, Lymphoid cells and Myeloid cells, from the Immune Response in Silico (IRIS) database [13], [14]. We tested if these genes were overrepresented in our list of 1704 genes significantly associated with overall survival. We used the Fisher Exact test for every cell type for 3 different groups of genes separately: 1) all significantly associated with overall survival, 2) genes with significant HR>1 and, 3) genes with significant HR<1. Overall, 24 tests (8 cell types×3 gene groups) were performed and we report p-values adjusted for multiple testing with Bonferroni correction.

#### Gene overlaps

Significance of overlaps between two groups of genes (with A and B number of genes) selected from the same pool of C = 20,341 expressed genes was tested using the hypergeometric test. The expected number of overlapping genes was calculated as (A×B)/C.

## Results

### Association of overall survival with clinical risk factors

**Figure 1:**
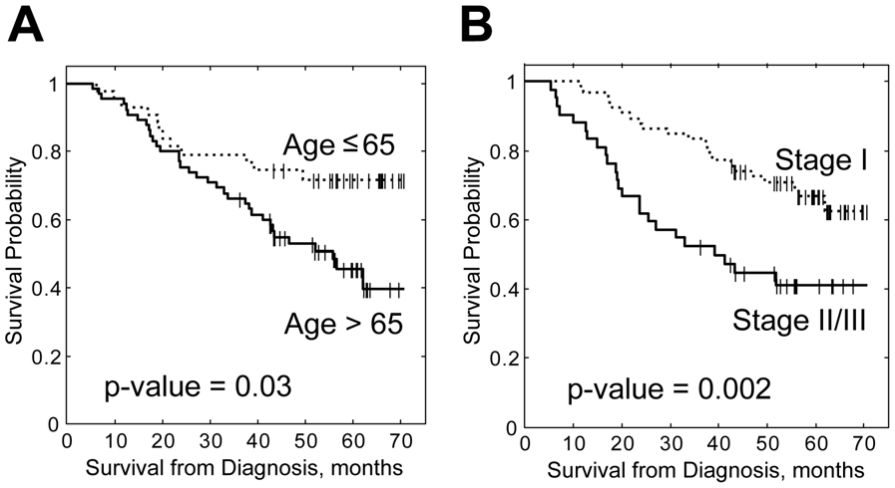
Kaplan-Meier curves for patients stratified by (A) Age and (B) Tumor Stage.

Various factors were tested for association with survival by univariate Cox regression, including tumor stage, age, gender, race, presence of COPD, tumor histology, tobacco use and adjuvant chemotherapy (**Table 1**). We found that two factors, advanced age and tumor stage, were significantly associated with survival with hazard ratios of 1.04 per year increase and 2.49 for tumor stage respectively. **Figure 1** demonstrates Kaplan-Meier curves for the two factors. When these two variables were tested together in a multivariate Cox regression model, both of them remained statistically significant (**Table 1**) indicating that they are independently associated with survival in our study population and that our data set conforms to predicted markers of clinical prognosis.

### Prediction of overall survival by a panel of PBMC genes

**Figure 2:**
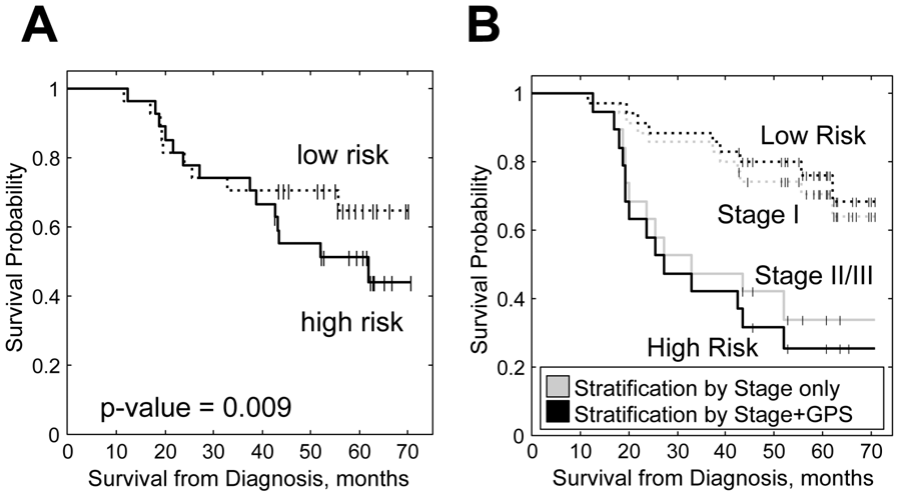
Performance of the Gene Predictive Score (GPS). **A.** Kaplan-Meier curves for patients from the test set stratified by the median of GPS calculated from expression of 26 genes. p-value for univariate Cox regression is shown. **B.** Kaplan-Meier curves comparing survival probabilities for patients from testing set stratified by Stage alone and combination of Stage and GPS with 35 patients in the lower risk group (number of stage I patients) and 19 higher risk group (number of stage II/III patients).

In order to identify and independently validate a compact gene panel, that could be used to predict overall survival, we divided our 108 samples into 2 randomly selected groups of 54 samples. We used a training set of 54 samples to select the prognostic gene panel, and then confirmed its utility on the remaining 54 of samples reserved as a test set. We applied multivariate Cox regression with L1 and L2 penalized estimations [8] to the training set, and identified 26 genes (**Table 2**) whose expression patterns when combined in a linear model, best predicted the observed survival data. For each subject, this model provides a Gene Prognostic Score (GPS) that is calculated as a linear combination of the expression values of the 26 prognostic genes. The GPS assigned to each patient was found to be significantly associated with his survival for the 54 subjects in the training set with *P* = 3×10^−5^. The performance was confirmed on the validation set (*P* = 0.009) demonstrating that the PBMC derived GPS is a statistically significant predictor of overall survival on new patients. Kaplan-Meier curves for patients from the validation set and their assignments into either a high-risk or low-risk category based on the median GPS are shown in **Figure 2A**. These studies show that PBMC expression levels for the 26 gene probes developed on our training set could also successfully predict survival in the validation set and, by extension, on any new patients.

**Table 2:** The 26 gene probes used in a calculation of the gene predictive score (GPS).

#	Gene	Symbol	c	c^norm^	*P*
1	coronin 6	CORO6	2.51	0.39	0.02
2	PREDICTED: similar to Nuclear protein 1 (prot p8)	LOC650200	−2.21	−0.38	0.001
3	PM1-EN0060-201000-002-c07 EN0060 cDNA	BF846242	−4.26	−0.37	0.01
4	transcription elongation factor A (SII)-like 4	TCEAL4	−1.08	−0.31	3×10^−5^
5	matrix metallopeptidase 1 (interstitial collagenase)	MMP1	0.82	0.22	0.02
6	cDNA clone IMAGE:6621749 5	BU854460	2.22	0.2	0.01
7	family with sequence similarity 20, member A	FAM20A	0.5	0.2	0.0003
8	T cell antigen receptor alpha chain	TCRVA2	−0.48	−0.19	0.0001
9	thioesterase domain containing 1	THEDC1	1.34	0.18	0.01
10	cDNA clone IMAGE:3643602 3	BF194881	1.55	0.16	0.05
11	glial high affinity glutamate transporter	SLC1A3	0.18	0.12	0.05
12	ADAM metallopeptidase with thrombospondin type 1 motif, 2	ADAMTS2	0.73	0.12	0.01
13	low density lipoprotein receptor-related protein 8	LRP8	0.55	0.12	0.03
14	zinc finger protein 662	ZNF662	−0.69	−0.11	0.01
15	secretin	SCT	−0.51	−0.09	0.01
16	C-type lectin domain family 4, member C	CLEC4C	−0.15	−0.08	0.02
17	InaD-like (Drosophila)	INADL	−0.37	−0.08	0.001
18	cyclin E2	CCNE2	0.36	0.08	0.001
19	kinesin family member 15	KIF15	0.19	0.06	0.004
20	cut-like 2 (Drosophila)	CUTL2	−0.15	−0.05	0.01
21	argininosuccinate synthetase	ASS	0.19	0.04	0.03
22	chromosome 5 open reading frame 20	C5orf20	−0.08	−0.03	0.005
23	tetraspanin 14	TSPAN14	0.08	0.03	0.01
24	PREDICTED: similar to zinc finger protein 114	LOC390372	−0.19	−0.02	0.1
25	C-type lectin domain family 4, member C	CLEC4C	−0.05	−0.02	0.08
26	complement component 4 binding protein, beta	C4BPB	−0.04	−0.01	0.01

c is a regression coefficient for the probe expression. c^norm^ is a c normalized over average expression among 26 probes to show relative contribution of the gene to the final GPS and is used to rank the genes. Regression coefficient is indicative of a hazard ratio for a gene: if c>0, then HR>1, if c<0, then HR<1. *P* shows univariate cox regression p-value for the gene when all 108 samples are used.

### Gene expression and tumor stage data are independent predictors of survival

We further assessed whether the GPS provided additional value to the clinical risk factors for survival we had tested. Of these, only age and tumor stage were found to have significant prognostic values for our data set. Although age was significantly associated with survival in analysis using all 108 patients' information (**Table1**), it did not have significant prognostic value when applied to the test set of 54 samples (*P* = 0.34). For this reason, we did not include the age variable in the following analyses.

Based on the data from the validation set, the stage predictor alone generated a hazard ratio of 3.0 (*P* = 0.0095, 95% CI of 1.3 to 6.9). When used in the multivariate Cox regression model, both Stage and GPS were significant (*P* = 0.004 for stage and *P* = 0.003 for GPS), indicating that the two variables are independent predictors of overall survival and that using them together should increase prognostic power. To assess this increase, we then compared hazard ratios for stage alone to the hazard ratios for the combined Stage+GPS factors. Stage alone split patients into 19 higher risk patients (the number of Stage II/III cases) and 35 lower risk patients (the number of Stage I cases).

.The combined Stage+GPS predictor resulted in a significant hazard ratio of 4.9 (*P* = 0.0003, 95% CI of 2.1 to 11.4) between high and low risk groups, an increase compared to the hazard ratio shown by Stage predictor alone (HR = 3.0) or GPS predictor alone (HR = 1.9). This is illustrated by the Kaplan-Meier curves (**Figure 2B**).

Alternative quantitation of the ability of the gene expression score to improve prediction of survival was performed by determining the concordance index for the Cox regression model for stage alone: 0.63 (0.56–7.0), GPS alone: 0.57 (0.51–0.62), and stage+GPS: 0.69 (0.62–0.76). The increase in concordance index along with the evidence of the independence of the two factors supports the utility of using both the stage and gene expression scores to determine probability of survival compared to using stage only. This result demonstrates that a gene expression score can add prognostic information to the traditional tumor stage variable.

Adding the GPS factor to the classification by stage resulted in the reassignment of 6 patients between high and low risk groups. Three of the 6 with stage I NSCLC (one IA and two IB patients) were reassigned to the high risk group. Two of these patients actually died within 18 months (p1240, stage IB) and 43 months (p1183, stage IA) of the sample collection. The additional 1B patient (p1246) remained alive for at least 65 months. This individual was one of the youngest patients in our cohort (only 47 compared to 45 year minimum age). Inclusion of the age factor in the model might have altered assignment to the high risk group. The 3 other patients had stage II NSCLC. They were classified by stage as high risk and then reassigned to the low risk group when adding the GPS factor. All three of the patients (p1589, p1561 and p1445) were alive at the time of status assessment (44, 46 and 56 months correspondingly). These results support the utility of a combined GPS+stage model for more accurate estimation of prognosis.

### PBMC gene functions associated with overall survival

In order to determine whether specific functions or pathways were represented in the PBMC gene expression patterns associated with survival, we used univariate Cox regression to identify the genes that were significantly associated with outcome using all 108 patient samples. We found 1704 probes that showed significance at *P*<0.05 and screened those probes for enriched functions and pathways using both DAVID and GSEA software. This analysis revealed several highly significant functional categories with enrichment of 2 fold or more (**Table S2**). In particular, a list of 32 ribosomal structure and function-related genes (enrichment of 2.3) had higher expression in patients with better survival, while cell cycle genes in general (enrichment of 2.3) and specifically M phase genes (enrichment of 2.2) were predominantly expressed at lower levels in the patients with better survival.

### Immune cell types associated with survival

Since gene expression was analyzed on PBMC mRNA, we determined whether the genes significantly associated with survival were also specific to a certain immune cell type as defined by the Immune Response in Silico (IRIS) database [13], [14]. Of the 669 immune cell-specific genes in our dataset, 79 were found to be associated with overall survival (**Table 3**). We found a significant enrichment of T-cell specific genes (23 altogether, 8 fold overrepresentation, *P* = 2×10^−8^), with 15 of the 23 genes having HR>1 indicating a significant correlation between lower levels of expression and improved survival. We also found that lower expression of 21 myeloid specific genes (enrichment of 2 fold, *P* = 0.048) was associated with improved overall survival. Detailed lists of genes associated with each cell type and corresponding enrichments and p-values are listed in **Table S3**.

**Table 3:** Number of unique cell-type specific genes in a list of 1704 probes significantly associated with survival.

IRIS Cell type	HR>1(of 629)	HR<1(of 821)	Not significant(of 13489)
T Cell	**15** *	8	33
B Cell	2	1	59
NK Cell	1	0	11
Dendritic Cell	2	3	41
Monocyte	6	1	45
Neutrophil	2	0	31
Lymphoid	8	9	149
Myeloid	**21** *	0	221

Table shows numbers of unique genes.

* = significantly overrepresented (Bonferroni corrected *P*<0.05). For more details reference Table S3.

### Tumor removal changes expression of survival-associated genes

**Figure 3:**
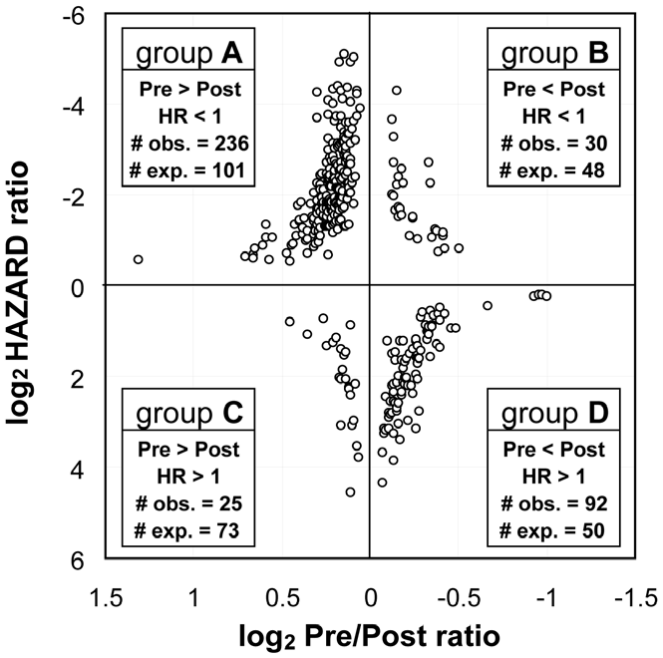
Characterization of genes whose expression changes after tumor removal and are also associated with survival. # obs. = observed number of genes; # exp. = expected number of genes by chance; Pre = expression in pre-surgery sample; Post = expression in post-surgery sample; HR = Hazard Ratio for high vs low expression of the gene.

We previously showed that PBMC gene expression in the presence of a NSCLC is significantly altered in post-surgery PBMC samples [5], [6]. We found that the expression of more than 20% (383) of the 1704 “survival” genes screened in this study was also significantly changed by the surgical removal of the lung tumor (3485 genes with p-value<0.05 by paired t-test, FDR<20%). This is a statistically significant number compared to the 292 gene overlap expected by chance (*P* = 10^−9^ by hypergeometric test). We identified 4 classes of outcome-associated genes (**Figure 3**) that change expression levels post-surgery. Two of the groups of genes (A and D) were significantly overrepresented and we describe these 2 classes. The largest group (group A) of 236 genes have HR<1 (better survival for higher expression) and are downregulated in PBMC after tumor removal. The second group (group D) includes 92 genes with HR>1 (poorer survival with higher expression) were upregulated in PBMC after tumor removal. These prognostic genes, that also change expression after the tumor is removed, could provide a new method for determining recurrence.

Functional enrichment analysis of group A genes shows strong overrepresentation of proteins associated with ribosomes, protein synthesis, mitochondrial function, translational factor activities and zinc finger proteins, characteristics consistent with PBMC being more active in response to the tumor presence and, apparently, better for survival. Group D shows functional enrichment only for genes associated with red cell function possibly indicating lower numbers of circulating erythroblasts in the presence of a tumor, but poorer survival with higher expression.

## Discussion

The ability to accurately identify patients with lung cancer that have a poor prognosis is particularly important for patients with Stage I disease who are known to vary significantly in likelihood of recurrence. Predicting which tumors are most likely to recur is critical to designing treatment after surgery, in particular for stage IA patients, which do not normally receive adjuvant chemotherapy. Currently, the most effective prognostic indicators are demographic characteristics (patient age, sex, smoking history) and clinical parameters (tumor size, stage and lymph node involvement). A number of gene expression studies on lung tumors that present prognostic algorithms were recently reviewed [4]. One analysis [15] trained and validated on publicly available data describes a 5-gene prognostic signature for all stages including stage I adenocarcinoma (but not squamous cell carcinoma). One issue with the tumor studies has been the lack of reproducibility, possibly because of differences in sample quality and tumor cell representation in the samples. Approaches using blood or serum to identify prognostic indicators avoid some of the problems associated with tumor variability and microRNA expression profiles in serum or plasma have been applied to both NSCLC diagnostic [16], [17] and prognostic studies by two groups [18], [19]. Hu et al, [19] using serum from 243 patients, found a prognostic set of 4 miRNAs which, when trained on half the patients could predict survival on the remaining half. Both groups suggest that their findings will allow separation of low from high risk patients, improving prognosis by stage alone.

Interactions between a tumor and the immune system have long been a subject of interest in cancer biology [20]. We previously found that PBMC gene expression signatures could distinguish patients with lung cancer from patients with non-malignant lung diseases. The studies presented here demonstrate that gene expression signatures in the peripheral immune cells of NSCLC patients also contain information that is correlated with survival and, like signatures obtained from lung tumors, can improve clinical predictors of outcome. In our study, we identify a panel of 26 gene probes whose expression patterns are significantly associated with survival and this information is independent of information provided by tumor stage. We also show that some genes correlated with prognosis are immune cell lineage specific. The most striking changes were in the myeloid specific genes which show an inverse correlation between expression and survival. As shown in **Table S3**, 29 of the 30 genes specifically associated with monocytes, neutrophils, or “myeloid cells”, have increases in expression in patients with decreased survival times. These data are consistent with observations that an increase in immature myeloid cell populations in the blood and in tumors are associated with advanced cancers and poor prognosis, likely due to ability of these cells to promote angiogenesis and to suppress anti-tumor immune responses [21], [22], [23], [24]. High numbers of neutrophils have been shown to be predictors of poor prognosis in melanoma, ovarian cancer and head and neck cancer [25]. Prognosis-associated T-cell genes also have predominantly higher hazard ratios but the mechanism by which these genes affect patient survival is not yet clear.

A significant number of PBMC genes that change in expression after the removal of a tumor are also associated with prognosis. The largest class (group A, **Figure 3**) includes genes which are expressed at higher levels in the presence of a tumor compared to samples from the same patient taken after the tumor removal and whose higher expression indicates better survival. The most differentially expressed gene in this group is the immune response gene *CXCR4*, important for lymphocyte trafficking [26], [27]. Its ligand, *SDF-1*/*CXCL12*, is a key factor directing dendritic cell migration associated with an adaptive immune response [27], [28] In contrast, another large group of genes (group D) show the opposite behavior having high hazard ratios indicating poor survival function and lower levels in the presence of a tumor. DAVID analysis of this gene class shows enrichment for genes significantly associated with functions of oxygenation, hypoxia and iron binding (*HGBD*, *HGB1*, *HGB2*) and erythroid associated factor (*ERAF*). These are presumably constituents of the erythroblasts known to co-purify with the PBMC [29], [30]. Lower expression of these genes in the presence of the tumor may indicate that erythroblast gene expression (or erythroblast numbers) is repressed in agreement with other studies, as poor oxygenation and anemia have been previously associated with lung cancer [31], [32]. Relatively higher expression of these genes in patients with poorer survival could be due to the increased presence of erythroblasts in response to the deteriorating oxygenation associated with more serious disease. Overall, the significant overrepresentation of genes from groups A and D suggests that measuring the change of those genes between paired pre- vs post- surgery samples from the same patient, might reflect the immune system's ability to respond to the tumor, and may provide added information for outcome prediction by comparing samples collected before with samples collected after tumor removal. In addition, genes from those groups may be candidates to reassess cancer recurrence probabilities in regular post-surgery blood samples.

The use of combined prognostic factors including clinical stage, tumor gene expression signature and PBMC gene signatures could provide additional guidance for prescribing adjuvant chemotherapy for early stage cancers that presently would not otherwise be treated after surgery. We find that the gene expression from patient PBMC is an independent predictor of survival, and that when PBMC gene expression is combined with information from cancer stage, the hazard ratio from stage alone is increased from 3.0 to 4.9. The limited size of the dataset has not permitted testing for prognosis within a single cancer stage, but we find that two stage I patients are correctly reassigned to the high risk group by combining the GPS blood signature with stage based prognosis.

The evolution of a malignant tumor includes interactions among cancer cells and signaling with other cells in the tumor microenvironment including immune cells. Our studies suggest that these interactions are not only localized to the tumor site but also extend to the peripheral immune system and that information related to prognosis or recurrence is present in the gene expression profiles of the peripheral immune cells. In contrast to tumor-based prognostic signatures that reflect malignancy, PBMC based prognostic signatures reflect the type and strength of the immune response to the tumor presence. It is likely that by combining clinical characteristics for survival derived from tumors at the time of surgery with repeated assessments of PBMC derived GPS data, a more robust algorithm for predicting recurrence can be developed that can be reassessed at various intervals after surgery. In addition, the genes of immune cells associated with patient outcomes might be targets for clinical manipulation to alter key gene expression in order to potentiate the anti-tumor immune response and help to reduce the incidence of recurrence.

While additional studies with larger numbers of racially diverse patients need to be carried out, the present study demonstrates that statistically significant outcome information can be detected in the gene expression patterns of PBMC from NSCLC patients. Efforts to collect the appropriate samples to address these issues are ongoing.

## Supporting Information

Figure S1 **Distribution of p-values for applying model fit on training set to testing set samples.** To test the generality of our approach, we performed 100 random selections of training/testing sets to estimate the possibility that our result can be accounted by a fortuitous selection of patients. Based on distribution of p-values that show significance of performance on the test set, we saw 39% of the test sets reached a statistical significance of *P*<0.05. This is an enrichment of 7.8 fold over a random p-value distribution (*P* = 4×10^−9^, Fisher exact test).
(TIF)

Table S1 **Demographics and survival data for patients.** S = current smoker (n = 15), Q = quit smoking (n = 23 with <1 year before surgery, n = 64 with ≥1 year before surgery), N = never smoker (6), C = Caucasian, AA = African American, M = male, F = female, y = yes, n = no, un = unknown, AD = adenocarcinoma, LSCC = lung squamous cell carcinoma, NSCLC = non-small cell lung cancer, a = alive, d = deceased.
(XLS)

Table S2 **Enriched annotation categories in the list of genes significantly associated with survival.** Results are based on univariate cox proportional hazard model. E = enrichment, Sensitivity shows how many of genes are in the list/how many total known (resulting percentage), FDR = false discovery rate from the DAVID software. HR = hazard ratio, and <1 indicates number of genes with higher expression in patients associated with better survival, while >1 indicates number of genes with higher expression in patients associated with poor survival. All results showed FDR<25% by GSEA analysis.
(XLS)

Table S3 **Expanded** **Table 3** **showing unique cell-type specific genes.** The table shows number of genes in a list of 1704 probes (1450 unique genes) significantly associated with survival. ‘+’ indicates genes with HR>1 (higher expression = worse survival). ‘-’ indicates genes with HR<1 (higher expression = better survival). S = number of significant genes specific to the cell type. NS = number of non-significant genes specific to the cell-type. E = enrichment of the cell-type specific genes among all significant genes. *p* = right-tail Fisher Exact Test nominal p-values with Bonferroni corrected p-values (24 tests = 8 cell types×3 gene sets) shown in parenthesis.
(XLS)
