# Supplementary figures and images for: Peripheral Immune Cell Gene Expression Predicts Survival of Patients with Non-Small Cell Lung Cancer
Source: PLoS One. 2012 Mar 29;7(3):e34392. doi: 10.1371/journal.pone.0034392 (PMC3315526; doi:10.1371/journal.pone.0034392)

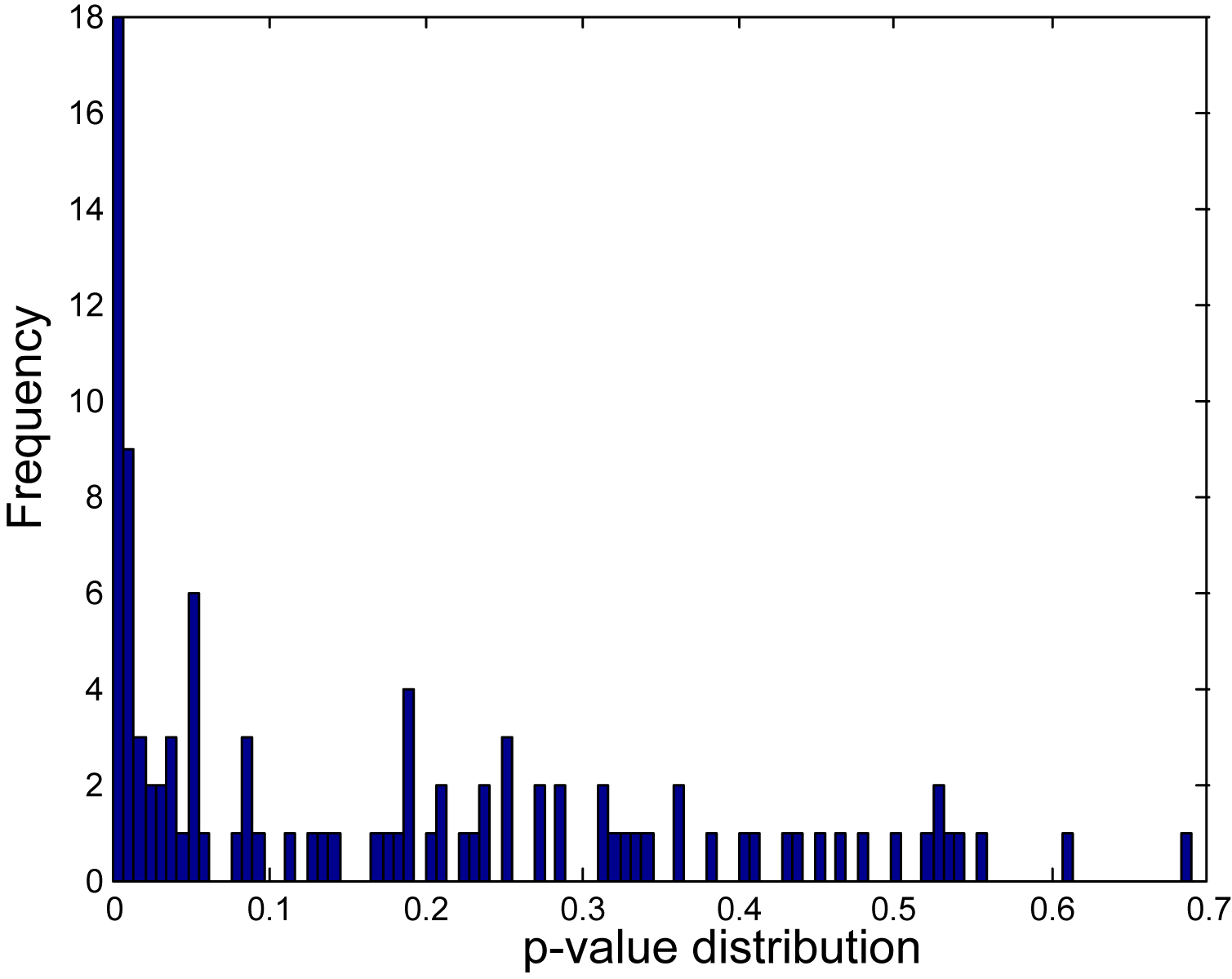

Supplement: Figure S1 — Distribution of p-values for applying model fit on training set to testing set samples. To test the generality of our approach, we performed 100 random selections of training/testing sets to estimate the possibility that our result can be accounted by a fortuitous selection of patients. Based on distribution of p-values that show significance of performance on the test set, we saw 39% of the test sets reached a statistical significance of P<0.05. This is an enrichment of 7.8 fold over a random p-value distribution (P = 4×10^−9^, Fisher exact test). (TIF) [file pone.0034392.s001.tif]
